# A novel parasternal transthoracic echocardiographic window for detecting coronary ostial dilation after modified Bentall surgery

**DOI:** 10.1186/1476-7120-11-14

**Published:** 2013-05-20

**Authors:** Austin Chin Chwan Ng, Dianna Hanzek, Leonard Kritharides, John Yiannikas

**Affiliations:** 1 Cardiology Department, Concord Hospital, The University of Sydney, Hospital Road, Concord 2139, NSW, Australia

**Keywords:** Coronary artery imaging, Aneurysm, Aortic operation, Computed tomography, Transthoracic echocardiography

## Abstract

**Background:**

During the modified Bentall surgery (aortic root replacement), a cuff of native aorta is implanted, together with the coronary ostium, into the aortic graft. Multi-detector computed tomography (MDCT) imaging can accurately assess the coronary ostial anastomosis site post-surgery. In this study, we assessed the feasibility of imaging the coronary ostial anastomosis site using transthoracic echocardiography (TTE).

**Methods:**

Patients (n = 14, mean age 65 ± 12 years, 79% males) with previous Bentall surgery underwent TTE study, with MDCT (64-slice) as the reference standard. TTE used conventional and novel acoustic windows to interrogate the coronary ostia.

**Results:**

All coronary ostia (n = 28) were well-visualized with MDCT. The optimum TTE acoustic window for visualizing the coronary ostia was a superiorly positioned parasternal short-axis view with the probe tilted towards the left shoulder, medially angulated for the right coronary artery ostia (RCAos) and laterally angulated for the left main coronary artery (LMAos). In this off-axis position, 10 (71%) LMAos and 13 (93%) RCAos could be visualized. In the conventional parasternal views, only 5 (36%) RCAos and no LMAos could be visualized. TTE underestimated the diameter of the LMAos (10.0 ± 2.4 mm TTE vs. 13.4 ± 2.7 mm MDCT, p = 0.007), but was similar to MDCT for the RCAos (9.8 ± 3.1 mm TTE vs. 11.1 ± 3.2 mm MDCT, p = 0.10).

**Conclusions:**

We report a novel TTE acoustic window to image the coronary ostia of post-Bentall surgery patients. Although TTE underestimates the left coronary ostium size, recognition of the ostial dilation with TTE appears feasible in most patients. Those that cannot be imaged will require alternative imaging modality such as MDCT.

## Background

The incidence of thoracic aortic aneurysm is approximately 4.5 per 100,000 people, of which 60% predominantly affects the supravalvular aorta (aortic root and/or ascending thoracic aorta) [1,2]. The patient with a large aortic aneurysm faces disease progression and an increased risk of dissection or rupture [3], and early surgical intervention is recommended.

The Bentall procedure [4], coupled with the use of the open button technique (modified Bentall procedure) [5], is the current recommended therapy for patients requiring surgical intervention for supravalvular aortic aneurysm (sparing the aortic valve in patients without associated aortic regurgitation) [2]. There have been only a few detailed studies during long-term follow-up on the ostial anastomoses site using modern imaging techniques [6]. There are occasional reports of apparent ostial ectasia [7], the significance of which is uncertain. Current multi-detector computed tomography (MDCT) have sufficient temporal and spatial resolution to serve as an alternative to invasive coronary angiography, particularly with respect to assessing disease of proximal coronary segments [8,9]. We previously reported on its potential role in assessing the coronary ostial anastomotic site of patients who have undergone the modified Bentall surgery and found coronary ostial dilation to be typical after surgery [10]. Serial monitoring with MDCT may not be feasible due to risk of radiation exposure. Transthoracic echocardiogram (TTE), on the other hand, is ideal due to its non-invasive nature. However, the lack of echocardiographic reports on coronary ostial dilation in these patients suggests that conventional echocardiography acoustic windows may not be adequate in assessing the coronary ostial anastomotic site in this specific population.

The present study reports a novel TTE acoustic window that appears to be superior to conventional acoustic windows in assessing the coronary ostial dilation of patients who had undergone the modified Bentall operation, with dual-source 64-slice MDCT imaging as the reference standard.

## Methods

### Patient population

Patients who had prior aortic root replacement with the Bentall’s technique were recruited over a period of 15 months. The referrals for imaging were all based on clinical review and recommendation by patients’ treating physicians as part of their long-term follow-up. Detailed background history was obtained from each patient and further corroborated with the patient’s medical record. Clinical information collected included indications for surgery, original pathology, comorbidites, original operation report, and associated histopathology reports.

### Surgical procedure

The original Bentall procedure and its subsequent modifications have been published previously [4,5]. In brief, Bentall et al. used a wrap/inclusion technique whereby the coronary ostia were not excised from the native aorta but sutured onto side-holes of the prosthetic aorta. Current approaches commonly use the ‘button’ technique with end-to-side re-implantation of the coronary ostia with a surrounding ‘button’ or cuff of native aortic tissue directly onto the aortic graft (modified Bentall operation). In this series, all patients underwent the modified Bentall procedure.

### Imaging modalities

Standard two-dimensional and Doppler TTE was performed by an experienced sonographer using commercially available transducer and equipment (M3S probe, Vivid7, GE, Horten, Norway). Echocardiograms were recorded and stored in a digital database and then analysed offline using commercially available software (GE EchoPac 7.0.1, Horten, Norway). Echocardiogram was performed with patients in the left lateral decubitus position. TTE used conventional and novel acoustic windows to interrogate the coronary ostia. Conventional acoustic windows concentrated on the parasternal short-axis views whilst several off-axis views were attempted to determine the optimum TTE acoustic window for visualizing the coronary ostia. The coronary ostial diameter was measured using electronic calliper at the coronary ostial-aortic graft junction from the optimum acoustic window.

All patients also underwent MDCT imaging using a dual-source 64-slice scanner (Siemens Medical Solutions, Forchheim, Germany). The scanner parameters have been previously published [8]. 100 ml of contrast (Omnipaque 350; Amersham Health, Princeton, USA) followed by 81 ml of saline flush was injected continuously at 3.5 ml/s. Bolus tracking (when aortic root image density value exceeded 100 HU) was used to initiate the scan after 8 seconds of monitoring delay. Axial images were reconstructed at 0.6 mm slice thickness and 0.3 mm increment, using retrospective electrograph gating. A medium-soft convolution kernel was used (B25F). Nitroglycerine was not used for the study cohort to eliminate any potential confounder when measuring coronary artery diameters. None of our patients required beta-blockers during their scans.

The diameters of the coronary vessels on CT scan were assessed offline using commercially available software (Syngo® Circulation, Siemens Medical Solutions, Erlangen, Germany). Measurements were performed from the ‘best diastole’ data set. The coronary ostial diameter was measured using electronic calliper at the coronary ostial-aortic graft junction. 0.6 mm multi-planar reconstructions were manipulated to obtain an image perpendicular to the aortic root at the level of the coronary ostium that showed the greatest ostial dilation on visual assessment. A soft tissue window was used (600/200).

### Ethics

The study complies with the Declaration of Helsinki and was approved by the institution (Concord Hospital) Ethics Committee on Human Research (CH62/6/2008-059).

### Statistics

All continuous variables are expressed as mean ± standard deviation. Mean coronary ostial diameters obtained from TTE versus MDCT were compared using paired *t* tests. Analysis was performed using PRISM 4.03 (GraphPad Software, Inc.). A two-tailed probability value <0.05 was considered statistically significant.

## Results

### Clinical characteristics and operative details

A total of 14 patients (mean age 65 ± 12 years; 11 males) underwent both TTE and MDCT imaging studies to assess the thoracic aorta (Table 1). Comorbidities included hypertension (n = 12) and hyperlipidemia (n = 8). Five patients were ex-smokers and none had diabetes. Four patients were found to be in rate-controlled atrial fibrillation at time of their MDCT scans. All had prior composite modified Bentall procedure replacing aortic valve and aortic root, and the button technique to re-implant the native coronary arteries to the aortic graft. One patient also had a hemi-arch replacement for ascending aortic aneurysm that extended into the aortic arch. All patients had severe pre-operative aortic root dilation (mean diameter by echocardiogram 51.6 ± 4.3 mm) and all underwent elective surgery apart from one patient who presented with spontaneous dissection of ascending aorta requiring urgent surgery. Three patients required coronary artery bypass grafting at the time of their root replacement. Six patients had pathologically confirmed bicuspid aortic valve, and most had evidence of aortic valve incompetence. One patient (no. 6) had an aberrant left circumflex coronary artery arising from the right coronary artery that occluded post-surgery, resulting in myocardial infarction.

**Table 1:** Individual patient MDCT and echocardiogram characteristics

**No.**	**Original pathology**	**Time to MDCT (months)**	**Diameters (MDCT) **^**a**^		**Time to echocardiogram (months)**	**Visible on TTE **^**b**^		**Diameters (TTE)**	
			**LMA ostium**	**RCA ostium**		**PS off-axis view (LMA ostium)**	**PS off-axis view (RCA ostium)**	**LMA ostium**	**RCA ostium**
1	AR. Dilated aortic root	121	14 mm	13 mm	143	Yes	Yes	10 mm	11 mm
2	Bicuspid AV. AR. Dilated aortic root.	106	14 mm	5 mm	125	No	Yes	-	7 mm
3	AR. Dilated aortic root.	57	13 mm	10 mm	75	Yes	Yes	11 mm	9 mm
4	Bicuspid AV. AR. Dilated aortic root.	107	16 mm	10 mm	113	Yes	Yes	14 mm	15 mm
5	Bicuspid AV. Dilated aortic root.	40	13 mm	9 mm	56	Yes	Yes	11 mm	8 mm
6	Bicuspid AV. Dilated aortic root.	62	10 mm	8 mm	75	Yes	Yes	6 mm	5 mm
7	AR. Dilated aortic root.	101	15 mm	13 mm	113	Yes	Yes	8 mm	9 mm
8	AR. Dilated aortic root.	83	11 mm	17 mm	94	Yes	Yes	9 mm	16 mm
9	Bicuspid AV. Dilated aortic root.	47	26 mm	10 mm	59	No	Yes	-	9 mm
10	AR. Dilated aortic root.	59	11 mm	14 mm	72	Yes	Yes	13 mm	13 mm
11	AR. Dilated aortic root.	115	12 mm	10 mm	124	Yes	Yes	8 mm	8 mm
12	AR. Dilated aortic root.	91	9 mm	14 mm	90	No	No	-	-
13	AR. Dilated aortic root.	75	10 mm	10 mm	84	No	Yes	-	8 mm
14	Bicuspid AV. AR. Dilated aortic root.	25	19 mm	15 mm	32	Yes	Yes	10 mm	10 mm

Abbreviations: *AR,* aortic regurgitation; *AV,* aortic valve; *MDCT,* multi-detector computed tomography; *TTE,* transthoracic echocardiography; *LMA,* left main coronary artery; *RCA,* right coronary artery; *PS off-axis view,* parasternal off-axis view (a superiorly positioned parasternal short-axis view).

^a^ Mean (±SD) heart rate during MDCT study for the cohort was 65 ± 9 beats per minute (bpm); all patients were in sinus rhythm during MDCT except for patient no. 1, 5, 7 and 11 who were in rate-controlled atrial fibrillation (maximum heart rates during study were 76, 68, 60 and 57 bpm, respectively).

^b^ In the standard TTE acoustic windows, none of the patients’ LMA ostia could be visualized and only five patients’ (no. 1, 7, 11, 12 and 14) RCA ostia could be visualized.

None of the patients required aortic valve or aorta re-intervention at time of the study, though one had a separate mitral valve replacement after developing severe mitral regurgitation six years after the original Bentall operation.

### Coronary ostial anastomotic site: different imaging characteristics

#### MDCT

The median interval between surgery and MDCT scan was 79 months (range: 25–121 months) (Table 1). The mean heart rate during MDCT scan for the cohort was 65 ± 9 beats per minute. The mean diameter of the aorta just distal to the aortic graft was 38.8 ± 5.7 mm. Five patients had aortic diameter >40 mm distal to the graft (mean 44.6 ± 3.8 mm). The mean coronary artery ostial diameter was 12.5 ± 4.0 mm (left main coronary artery ostia [LMAos] 13.8 ± 4.4 mm vs. right coronary artery ostia [RCAos] 11.3 ± 3.2 mm). Of the 28 coronary ostia, only 1 had a diameter <6 mm. All patients had at least one coronary ostial diameter ≥10 mm (93% of LMAos and 79% of RCAos).

#### TTE

**Figure 1:**
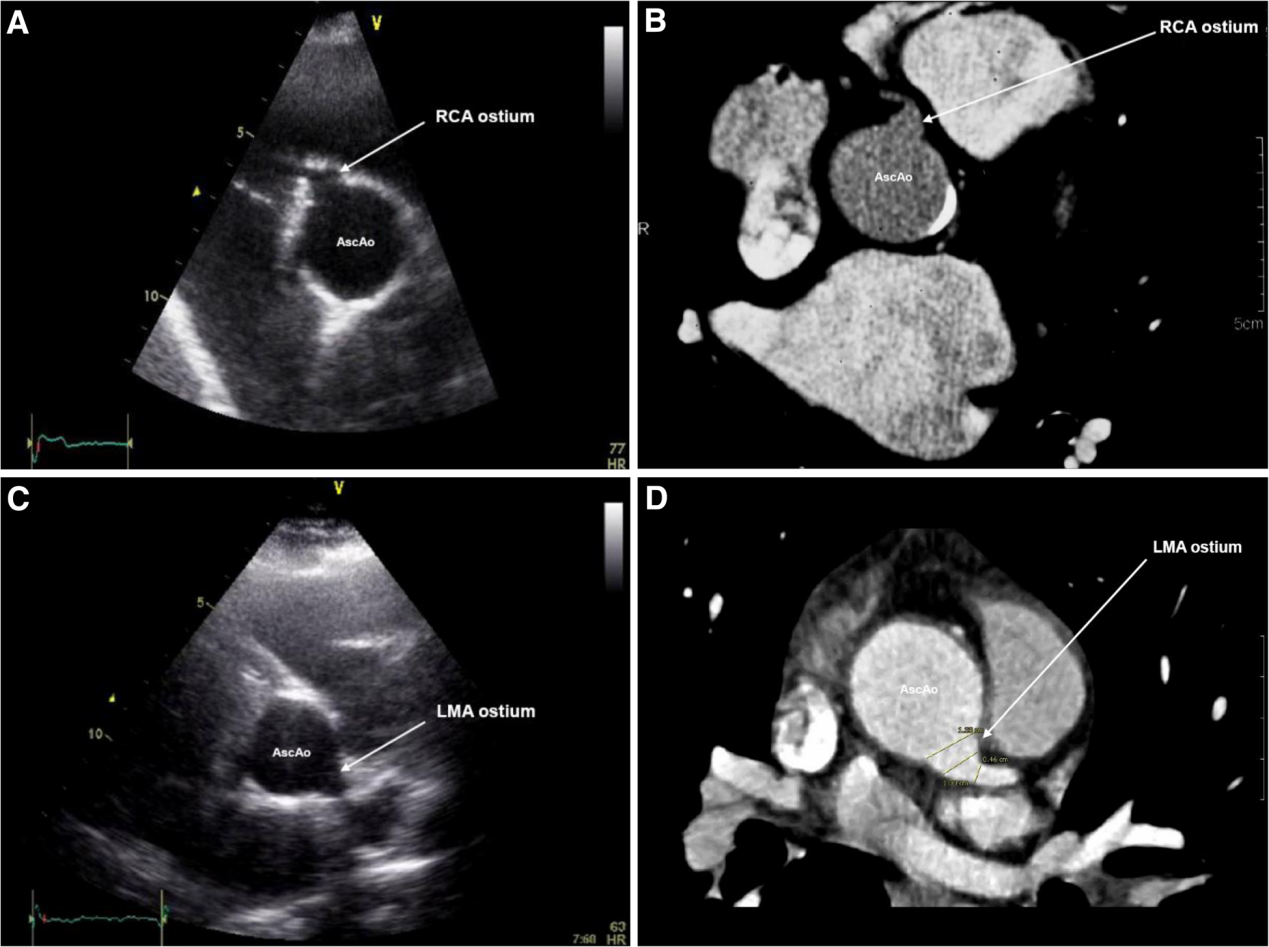
**Examples of imaging the coronary artery-aortic graft anastomotic site using transthoracic echocardiogram (TTE) and multi-detector computed tomography (MDCT). A**, an off-axis TTE acoustic window derived from the parasternal short-axis view of the right coronary artery (RCA) ostium of patient no.1 at the coronary-aortic graft anastomotic site (patient no.1) and **B**, visualization of the RCA using MDCT of the same patient (no.1); **C**, an off-axis TTE acoustic window derived from the parasternal short-axis view of the left main coronary artery (LMA) ostium of patient no.3 at the coronary-aortic graft anastomotic site and **D**, image of the LMA ostium using MDCT on the same patient (no.3). AscAo, ascending thoracic aorta. All vessel diameters were measured at the coronary ostial-aortic graft junction for both MDCT and TTE imaging techniques.

The median interval between surgery and TTE study was 87 months (range: 32–143 months). In the conventional parasternal views, only 5 (36%) RCAos and no LMAos could be visualized. Different off-axis views were then attempted to visualize the coronary anastomotic sites. The optimum TTE acoustic window for visualizing the coronary ostia was found to be in a superiorly positioned parasternal short-axis view with the probe tilted towards the left shoulder, medially angulated for the right coronary artery ostia (RCAos) and laterally angulated for the left main coronary artery (LMAos). In this off-axis position, 10 (71%) LMAos and 13 (93%) RCAos could be visualized (Figure 1). TTE underestimated the diameter of the LMAos (10.0 ± 2.4 mm on TTE vs. 13.4 ± 2.7 mm on MDCT, p = 0.007), and was similar to MDCT for the RCAos (9.8 ± 3.1 mm on TTE vs. 11.1 ± 3.2 mm on MDCT, p = 0.10).

## Discussion

We previously identified apparent dilation of the post-surgical coronary ostium in almost all patients who had undergone the modified Bentall’s operation some years earlier using a dual-source 64-slice MDCT [10]. The long-term clinical consequences of this coronary ostial dilation remain unexplored. The present study showed that whilst conventional TTE failed to adequately assessed the coronary ostia of these patients, an alternative novel TTE acoustic window was able to visualize most of the coronary ostia, thus providing a potentially suitable alternative non-invasive imaging tool to monitor the coronary ostial dilation long-term.

The normal diameter of native coronary vessels can be underestimated in post-mortem and conventional invasive angiographic studies [11-14]. These studies usually assess only the mid-portion of the proximal segment of the coronary vessels rather than the ostium (the aorto-coronary junction), and report diameters of 2–5 mm for the RCA and 4–7 mm for the LMA. Mean ostial diameters of 9.6 ± 1.8 mm (LMA) and 9.4 ± 2.2 mm (RCA) were reported in one study of normal subjects using a 16-slice MDCT scanner [12]. In contrast, the mean ostial diameter in the present study of post-Bentall operated patients was 12.5 ± 4.0 mm (LMAos 13.8 ± 4.4 mm vs. RCAos 11.3 ± 3.2 mm) using a 64-slice MDCT scanner.

Veira et al. reported a dilated appearance of the aortic-coronary artery anastomosis site of a patient immediately following composite aortic root and valve replacement using intraoperative transoesophageal echocardiogram [15], suggesting that the coronary ostial dilation could be an immediate consequence of the surgery. However, it is not known if the dilation can increase with time. Given that the residual aortic ‘button’ tissue may be similarly diseased as the explanted aortic aneurysm, continued longitudinal evaluation will help clarify whether the observed post-surgery coronary ostial dilation will increase in size over time.

**Figure 2:**
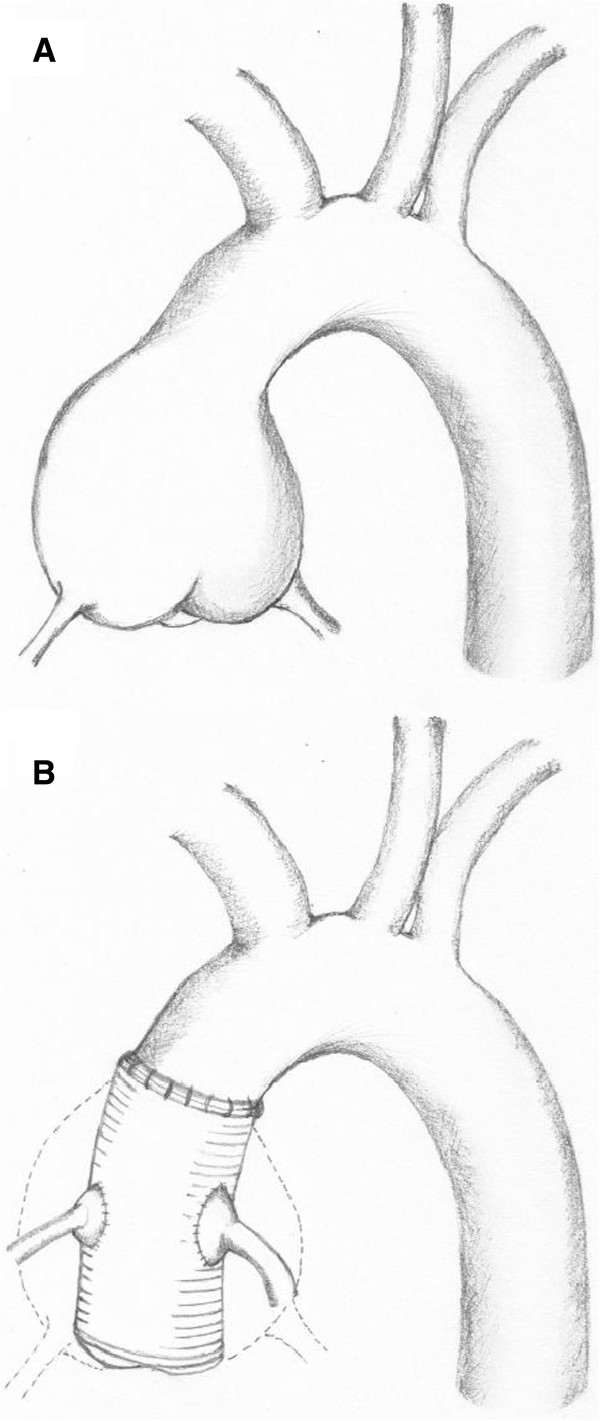
**Position of proximal coronary artery segment to the aorta before and after Bentall surgery.** Figure **A** shows the normal anatomy of the native coronary arteries arising from the coronary sinuses of the native aneurysmal ascending aorta before surgery. Figure **B** shows the mobilized native coronary arteries and the ‘button’ or cuff of native aortic tissue being re-implanted end-to-side to the prosthetic aortic graft post Bentall surgery. The ‘new’ coronary ostia-aorta anastomotic site is now positioned superiorly compared to the original anatomy.

However, if conventional TTE acoustic windows were used, none of the LMAos could be visualized and only a minority of the RCAos was seen. This was despite significant coronary ostia dilation in nearly all patients confirmed on MDCT. This would explain the lack of reported coronary ostia dilation using TTE in these patients. This is not unexpected given the altered anatomy of the coronary ostia-aortic junction post surgery. The button technique results in the explanted native coronary arteries being sutured onto side-holes of the prosthetic aorta in a position that is more superior to the original position of the native vessels (Figure 2). Hence, a more superiorly positioned parasternal short-axis view is required to visualize the coronary ostia of these patients, with the probe tilted towards the left shoulder, medially angulated for the RCAos and laterally angulated for the LMAos. In this novel view, we found the RCAos compares well to the MDCT measured diameters, whilst the LMAos’ diameters appeared underestimated on TTE compared to MDCT. The difference in measurements may be partially explained by the spatial resolution of the two imaging techniques. While a 64-slice MDCT scanner has a spatial resolution of 0.4 mm [16], two-dimensional echocardiography typically has a spatial resolution of 1.5 mm and variable depending on the frequency of the probe [17]. Thus, at these arterial diameters, the measurements obtained from MDCT will have greater accuracy than TTE. Although poor TTE acoustic window may further limit accurate measurements, the measured coronary ostia diameters on TTE in the present study were still large and should be adequate to allow long-term assessments for any signs of progression.

### Limitations

Although this is a small cohort study, and will require validation, we combined the images derived from MDCT to corroborate our findings from TTE. Most of the TTE were performed several months later than the MDCT. Future studies should address the issue of whether the coronary ostia dilation of these patients can change or progress with long-term imaging assessments. Although majority of the coronary ostia could be visualized on TTE using the proposed novel acoustic window, not all were observed. The TTE acoustic window may be limited in some post-thoracic surgery patients irrespective of the position of the probe. Those that cannot be observed on TTE may require MDCT to monitor their coronary vessels and aorta. We did not have a TTE control group. Our purpose was to show that conventional TTE acoustic windows are inadequate in visualizing the coronary ostial dilation of post-Bentall patients that were easily seen on MDCT. While our proposed novel TTE acoustic window allowed visualization of most of these patients’ coronary ostia-aortic junction, it is not known if the measured coronary ostial diameters will be significantly different from normal coronary vessels diameters measured using TTE.

## Conclusions

The significance of coronary ostial dilation in patients after modified Bentall surgery remains unclear. Long-term follow-up assessments using echocardiography as well as MDCT may help assess the impact of this finding on patient course and management. The present study described a novel transthoracic echocardiographic acoustic window that visualized the coronary ostia of most patients after Bentall surgery and confirmed similar dilated coronary ostia seen on MDCT. Although transthoracic echocardiography underestimates the size of the left main coronary ostium, recognition of the ostial dilation with transthoracic echocardiogram appears feasible in many patients and avoids the radiation exposure associated with MDCT. In those patients with inadequate echocardiographic acoustic window, using MDCT to image the coronary ostia of these patients post-surgery can be considered.

## Abbreviations

AscAo: Ascending thoracic aorta; AR: Aortic regurgitation; AV: Aortic valve; LMAos: Left main coronary artery ostia; MDCT: Multi-detector computed tomography; PS off-axis view: Parasternal off-axis view; RCAos: Right coronary artery ostia; TTE: Transthoracic echocardiography.

## Competing interest

We declare that we have no competing interest.

## Authors’ contributions

ACN designed the study, performed the main data collection, performed all data analyses and wrote the first draft of the report and subsequent revision with assistance from LK and JY. DH reviewed the data analyses, reviewed and revised the manuscript. LK reviewed the data analyses, reviewed and revised the manuscript. JY designed the study, reviewed the data analyses, reviewed and revised the manuscript. All authors read and approved the final manuscript.
